# Preparative Separation of Flavonoids from Goji Berries by Mixed-Mode Macroporous Adsorption Resins and Effect on Aβ-Expressing and Anti-Aging Genes

**DOI:** 10.3390/molecules25153511

**Published:** 2020-07-31

**Authors:** Jianfei Liu, Jiao Meng, Jinhao Du, Xiaofeng Liu, Qiaosheng Pu, Duolong Di, Chang Chen

**Affiliations:** 1 CAS Key Laboratory of Chemistry of Northwestern Plant Resources and Key Laboratory for Natural Medicine of Gansu Province, Lanzhou Institute of Chemical Physics, Chinese Academy of Sciences, Lanzhou 730000, China; jfliu@licp.cas.cn; 2 University of Chinese Academy of Sciences, Beijing 100049, China; 3 National Laboratory of Biomacromolecules, CAS Center for Excellence in Biomacromolecules, Institute of Biophysics, Chinese Academy of Sciences, Beijing 100101, China; mengjiao@ibp.ac.cn; 4 National Laboratory of Biomacromolecules, Institute of Biophysics, Chinese Academy of Sciences, Beijing 100101, China; djh_8122069@163.com (J.D.); 18009400938@163.com (X.L.); 5 State Key Laboratory of Applied Organic Chemistry, Key Laboratory of Nonferrous Metals Chemistry and Resources Utilization of Gansu Province, Department of Chemistry, Lanzhou University, Lanzhou 730000, China; puqs@lzu.edu.cn

**Keywords:** flavonoids, mixed-mode macroporous adsorption, resins, Goji berries, Aβ-expressing, anti-aging genes

## Abstract

Flavonoids are the main constituents of Goji berries and have good biological and pharmacological activities. The mixed-mode macroporous adsorption resins (MARs) for purification of flavonoids from Goji berries through computer-assisted calculation of the molecular size of flavonoids and the precise matching of MAR physical and chemical properties was firstly developed in the present study. Ten varieties of MARs with suitable molecular dimensions and polarities were used for investigating the adsorption/desorption behaviors of the flavonoids. Both AUKJ-1 and BWKX-1 showed higher separation efficiency than other MARs and then were mixed in different ratios to constitute a mixed-mode macroporous adsorption resin to obtain the optimal adsorption phase. Under optimal conditions, total flavonoid content of purified flavonoid (p-FLA) extract increased from 0.97% to 36.88% after one purification. The p-FLA extract from Goji berries significantly improved the expression of six genes with anti-aging effects and played an important role in aging-related Alzheimer’s disease by down-regulating Aβ expression.

## 1. Introduction

*Lycium barbarum* L. is known as the wolfberry or Goji berry. The fruits derived from *Lycium barbarum* L. have been consumed as food or medicine for more than 4000 years. Currently, China is the largest world producer with its 82,000 ha of cultivated land and 95,000 t of berries produced per year [1,2,3]. There are many instances in Chinese history in which people become healthier and longer-lived due to the tonic effect of Goji berries. The medicinal effect of Goji berries has long been recognized and appreciated by numerous medical scientists in the history of China. Traditionally, the herb is recommended for the nourishment of the liver and kidney and as an eye tonic [4,5]. Various scientific studies have reported that Goji berries possess numerous pharmacological properties, such as hypoglycemia, hypolipidemic, immunomodulatory, antioxidant, anti-aging, neuroprotective, and anti-Alzheimer’s disease activities [3,6]. The fruits of Goji berries contain diverse phytochemicals, like polyphenols, polysaccharides, flavonoids (FLAs), carotenoids, saponins, and polypeptides. FLAs are widespread secondary metabolites present in various plant-species and extensively used in food industries, cosmetics, medicines, and other fields. Major FLAs isolated from wolfberry include myricetin, rutin, quercetin, Kaempferol, quercitrin, nicotiflorin, 7-*O*-β-d-glucopyranosyl-rutin, isorhamnetin 3-*O*-rutinoside, hesperidin, Cerrone, alpinumisoflavone, and auriculasin, and their chemical structures are shown in Figure S1 (Supporting Information) [7,8,9,10]. The total flavonoid content ranges from 380 to 1600 μg/g with a slight difference between cultivars and production area. Rutin accounts for about 20–65% of the total flavonoid components. Recent studies have revealed the involvement of FLAs from Goji berries in many biological phenomena, such as hypocholesterolemic, antitussive, antioxidant, and vasodilatory activities [11,12,13]. Because of the wide range of biological activities of FLAs from Goji berries, these are extensively employed in the areas of medication, food, and health products [14]. However, very few studies had been focused on the isolation, separation, and purification of FLAs from Goji berries. In fact, the supernatant from the preparation of *Lycium barbarum* polysaccharide with water extraction and alcohol precipitation contains a large amount of FLAs, but it is often ignored or even discarded. This not only pollutes the environment but also wastes a lot of valuable resources. Therefore, we aimed at developing a simple, environmentally friendly, and efficient method to isolate FLAs from the supernatant.

Macroporous adsorption resins (MARs) are functional polymeric materials with better structural diversity, lower cost, better acid and alkali resistance, porous availability, higher surface area, environmental friendliness and longer lifetime than other adsorbents [15,16]. As powerful separating agents, MARs have numerous applications in various fields, such as chromatographic analysis, treatment, medical, wastewater disposal, and especially in the isolation and purification of active ingredients from different herbal extracts in traditional Chinese medicine [17,18,19,20]. Our group has been engaged in the application research of MAR for a long time [21,22,23,24,25]. We evaluated the adsorption properties of twenty-four kinds of MARs with lycopene oleoresin and screened out the best LX-68 resin from the dynamic adsorption/desorption experiments through a column packed with LX-68 resin; the best separation conditions were obtained, and the lycopene content in lycopene oleoresin increased 30.4-fold from 0.21% to 6.38%, with a recovery yield of 66.9% [26]. Considering that the functional group of the MAR is one of the most important key factors for separation efficiency, we synthesized a series of MARs with special functional groups including chloromethyl, amino, phenylamino, and ionic liquids through the Friedel Crafts and amination reaction and then used them for the separation of FLAs, epigallocatechin gallate (EGCG), and caffeine (CAF) from crude extracts of natural products, and obtained better separation efficiency and recovery [27]. Due to the broad-spectrum separation characteristics of MARs, the separation of compounds that possess similar structures by a single type MAR is difficult and impractical. We built mixed-mode resin separation technologies composed of two or more varieties of MARs to obtain a powerful separating agent and to separate bioactive components from crude extracts of herbal raw materials. However, to the best of our knowledge, there is currently no report on the isolation of FLAs from Goji berries by mixed-mode macroporous adsorption resins (mixed-mode MARs) [28,29].

Herein, the mixed-mode MARs for purification of flavonoids from Goji berries through computer-assisted calculation of the molecular size of FLAs and the precise matching of MAR physical and chemical properties was firstly developed. In the present study, rutin and myricetin, the two major compounds from Goji berries, were selected as a representative of FLAs. The molecular dimensions and polarities of both rutin and myricetin were calculated by HyperChem Release 8.0, and 10 different varieties of MARs with suitable molecular dimensions and polarities were utilized to investigate the adsorption/desorption behaviors of rutin and myricetin. AUKJ-1 and BWKX-1 showed higher separation efficiency than other MARs and were mixed in different ratios to constitute mixed-mode MARs to obtain the optimal adsorption phase. Then, the influences of initial FLA concentration, ethanol concentration, loading flow rate, and eluting flow rate were systematically optimized for the best purification effect. The mixed-mode MARs at a ratio of 2:1 showed the highest separation efficiency. In terms of enrichment and separation of actual FLAs of Goji berries from the supernatant by the preparation of *Lycium barbarum* polysaccharide, the content of purified FLAs (p-FLAs) increased from 0.97% to 36.88% in the crude extract samples. Then, we investigated the effect of the p-FLAs from Goji berries on Aβ-expressing and anti-aging genes; p-FLAs significantly improved the expression of six genes with anti-aging effects and played an important role in aging-related Alzheimer’s disease by down-regulating Aβ expression.

## 2. Result and Discussion

### 2.1. Screening of Optimum Resin

**Figure 1:**
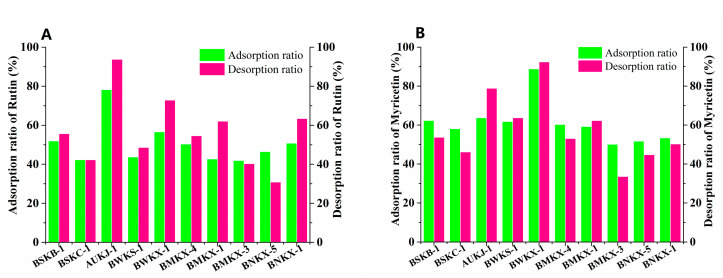
Adsorption/desorption ratios for rutin (**A**) and myricetin (**B**) on ten resins.

Flavonoid could adsorb on MARs through Van der Waals forces or hydrogen bonding due to its non-polar and polar groups. The pore size and polarity of macroporous adsorption resins are the main factors affecting the adsorption of flavonoids. A pore size of 2–6 times the molecular size is generally considered favorable for an adsorbate. Myricetin and rutin are the major flavonoid aglycone and flavonoid glycoside in Goji berries [30,31]. The molecular dimensions of myricetin and rutin are 1135 nm and 1382 nm as calculated by HyperChem Release 8.0., respectively. Thus, the favorable ranges of pore size for myricetin and rutin are 2270–6810 nm and 2764–8292 nm, respectively. Ten kinds of macroporous adsorption resins satisfying the above pore size requirements were selected for enrichment and separation of flavonoids form Goji berries, two of them with strong polarity (BSKB-1 and BSKC-1), three of them with medium polarity (BMKX-4, BMKX-1 and BMKX-3), three of them with weak polarity (AUKJ-1, BWKS-1, and BWKX-1), and the others with non-polarity (BNKX-5 and BNKX-1); the physical and chemical parameters are shown in Table S1 (Supporting Information). From the static adsorption/desorption studies, adsorption/desorption efficiencies of ten different varieties of MARs were described, as seen in Figure 1. Among the 10 resins, AUKJ-1, BWKS-1, and BWKX-1 showed better adsorption of rutin and myricetin in Goji berries than others; this is because the polarity of rutin and myricetin is weak. The most suitable separation of myricetin and rutin should be weak polar resin, according to the principle of polarity similarity of macroporous adsorption resins. However, BWKS-1 did not exhibit good adsorption rate for rutin and myricetin in the three resins, probably because its pore size was too large to exert a good screening effect. In addition, the specific surface area of AUKJ-1, BWKS-1, and BWKX-1 are 1326.9, 600.0, and 985.7 m^2^/g, in turn. The adsorption properties of these three resins for rutin and myricetin are positively correlated with their specific surface area; resin with large specific surface area has better adsorption capacity, indicating that the specific surface area of the resin is also an important factor affecting the adsorption effect of FLA. During the desorption process, all the MARs showed good desorption properties in the presence of 60% ethanol. The adsorption and desorption rates of BWKX-1 and AUKJ-1 for rutin and myricetin were 76.72%, 91.36%, and 88.70%, 92.36%, respectively. As shown in Figure S3 (Supporting Information), BWKX-1 and AUKJ-1 reached the adsorption equilibrium of rutin and myricetin in about 2 h, respectively, showing the strongest adsorption and desorption. All in all, the matching of the pore size of the adsorbent with the size of the adsorbate and suitable specific surface areas of MARs were considered to be the predominant factors affecting separation. Considering the preselection results, BWKX-1 and AUKJ-1 were chosen for the following study.

### 2.2. Separation of FLAs by Mixed-Mode Macroporous Adsorption Resins

**Figure 2:**
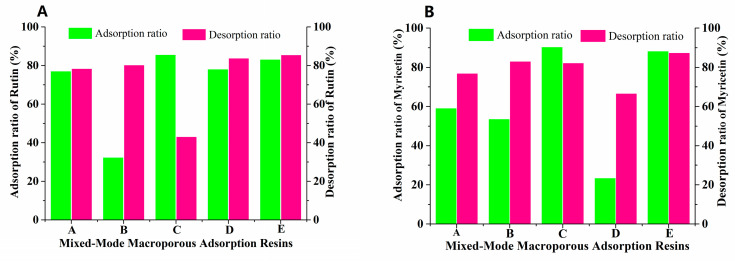
Adsorption/desorption ratios of rutin (**A**) and myricetin (**B**) on five different ratio resins (A–E: AUKJ-1 and BWKX-1 in 1:1, 1:1.5, 1.5:1, 1:2, 2:1 in turn).

From the wolfberry flavonoids currently found, we can see that they are mainly composed of flavonoid glycosides and aglycones, so it is difficult and impractical to separate these compounds with different physical and chemical properties by a single type of MAR [28]. For this reason, mixed-mode MARs with different proportions were screened to separate FLAs from the Goji berries. Based on the separation experiments of single MAR, the separation effect of FLA with two types of MARs mixed bed was studied. During this process, AUKJ-1 and BWKX-1 were mixed in different ratios (1:1, 1:1.5, 1.5:1, 1:2, 2:1) to constitute mixed-mode MARs with optimal adsorption properties, and to perform static adsorption/desorption studies. As seen in Figure 2, mixed-mode MAR E composed of AUKJ-1 and BWKX-1 in a 2:1 ratio showed the best adsorption and desorption properties for both rutin and myricetin, and they were also better than the separation performance of ten single resins, which meant that specific surface area and molecular exclusion effects between the adsorbent and the adsorbate could be perfectly coordinated. Each MAR possessed its own structural parameters, so the synergistic effect between different MARs occurred after forming a MARs mixed bed, which was the reason why the MARs mixed bed could improve separation efficiency [29].

### 2.3. Adsorption/Desorption Kinetics on Mixed-Mode MARs

**Figure 3:**
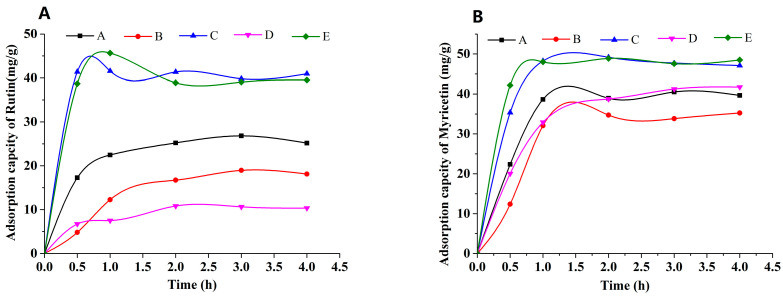
Adsorption capacity of rutin (**A**) and myricetin (**B**) on mixed-mode MARs (A–E: AUKJ-1 and BWKX-1 in 1:1, 1:1.5, 1.5:1, 1:2, 2:1 in turn).

**Figure 4:**
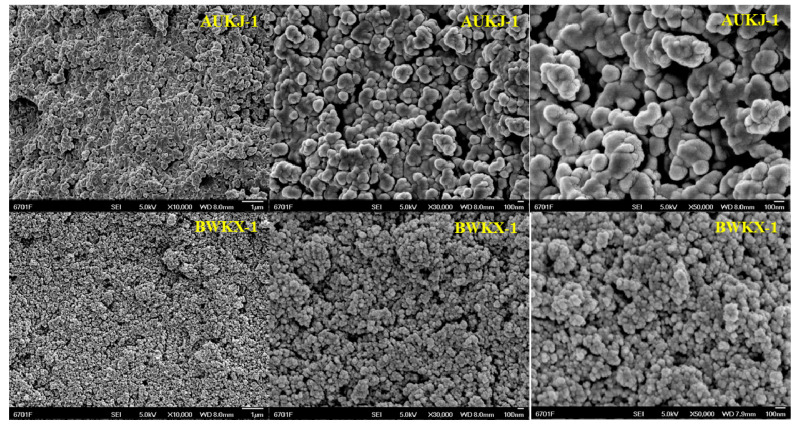
The SEM images of the internal structure of AUKJ-1 and BWKX-4 after grinding at a magnification of 10,000×, 30,000× and 50,000×.

The adsorption kinetic curves of rutin and myricetin on the mixed-mode MARs were obtained, as shown in Figure 3. Mixed-mode MAR E and C composed of AUKJ-1 and BWKX-1 in both 2:1 and 1.5:1 showed better adsorption properties for both rutin and myricetin. The adsorption capacity increased rapidly in the first 1 h due to rapid attachment of rutin to the macropore of mixed-mode MAR E and then increased slowly because of the diffusion of rutin from the macropore into the mesopore/micropore until reaching equilibrium after 4 h. In other words, the adsorption capacity of rutin on mixed-mode MAR E increased with the extension of adsorption time until equilibrium. Adsorption phenomenon of myricetin was the same as that of rutin. The adsorption processes of rutin and myricetin tended to balance in about 4 h, which indicated that the adsorption process was the slow adsorption type with mixed-mode MAR E (fast adsorption, *t* < 2 h; intermediate speed adsorption, 2 h < *t* < 3 h; and slow adsorption, *t* > 3 h) [29]. In short, the adsorption time was greater than 4 h to ensure complete adsorption. The slow adsorption was caused by the following reasons: the molecular sizes of rutin and myricetin are different (Figure S2, Supporting Information), and the pore size of mixed-mode MAR E composed of different resins is also heterogeneous. Hence, high intraparticle mass transfer resistance existed in the process of rutin and myricetin diffusing into the micropore of the mixed-mode MAR E. As shown in Figure 4, MARs possess many pores in different diameters. Many smaller pores existed in the inner face of the larger pores; these pores made MARs present properties that were obviously different from other materials. Due to their particularity in structure, the adsorption of MARs first took place on the macropores until all macropores nearly reached equilibrium, and then on mesopores and micropores in turn, until all accessible surfaces of MARs reached their adsorption equilibrium. The adsorption of MAR to organic compounds in aqueous solution was mainly achieved by Van der Waals forces, hydrogen bonding, hydrophobic interaction, and electrostatic interaction (including ionic bonding, covalent bonding, and coordination bonding). Because of the interlaced pore structure in MARs, there was the capillary effect during the adsorption process, which influenced the adsorption feature to a larger extent. The infiltrative solution played an important role in the capillary effect. According to the soakage principle, if the solution was infiltrated to MAR, capillary elevation would take place, and the capillary depression could be found clearly at the critical state of the interim from larger pore sizes to smaller pore sizes. This would result in significant fluctuations in the adsorption curve. Thus, the waves of adsorption capacity in the adsorption process of rutin and myricetin with mixed-mode MAR E could be attributed to the capillary effect Figure 3 [32]. As shown in Figure S3 (Supporting Information), the desorption capacity of the mixture of mixed-mode MAR E was the best; the desorption rates for rutin and myricetin were 34.37 mg/g and 44.04 mg/g, respectively.

### 2.4. Dynamic Adsorption/Desorption on a Mixed-Mode MARs

#### 2.4.1. Effect of Loading Concentration

In the process of dynamic adsorption and desorption, the loading concentration is an important factor affecting the adsorption/desorption capacities/ratios and purity. The results shown in Table S2 (Supporting Information) demonstrated that as the loading concentration increased, the content of rutin and myricetin also increased in the adsorption residue. The concentration of rutin and myricetin was the lowest when the feeding concentration was 400 g/L, which was 40.10 μg/mL and 6.91 μg/mL, respectively. In fact, when the loading concentration was 600 g/L, the content of rutin and myricetin in the adsorption residue was close to 400 g/L. Taking into account production efficiency and cost, the feeding concentration at 600 g/L was selected as the optimum.

#### 2.4.2. Effect of Loading Rate

Flow rate is also a noteworthy parameter affecting separation efficiency. If the flow rate is too high, some flavonoids will not be adsorbed on the macroporous resin, which will reduce the adsorption amount and purity of the product. Slow flow rate increases production costs and reduces production efficiency. As seen in Table S3 (Supporting Information), we can see the lowest concentrations of rutin (95.68 μg/mL) and myricetin (16.70 μg/mL) were obtained at the loading rate of 10 BV/h in the adsorption residue. Increasing the flow rate would significantly cause a large amount of flavonoids not to adsorb on the macroporous resin. Therefore, we chose 10 BV/h as the suitable loading rate.

#### 2.4.3. Effect of Ethanol Concentration

Eluents are known to trigger desorption processes by disrupting the interactions between the adsorbent and the adsorbate. When the flavonoids are completely adsorbed on the mixed-mode MARs, it is very important to select a suitable eluent. Goji berries contain a large amount of flavonoid glycosides (such as rutin, nicotiflorin, etc.) and flavonoid aglycones (such as myricetin, quercetin, etc.), and their physical and chemical properties determined that ethanol aqueous solution was the best elution system. In this paper, the effect of ethanol concentration for rutin and myricetin was investigated in order to obtain the best elution conditions. As can be seen from Table S4 (Supporting Information), the highest concentrations of rutin (503.03 μg/mL) and myricetin (549.25 μg/mL) in the desorption solution were obtained with 60% ethanol during desorption, indicating that 60% ethanol was the optimum composition for the desorption liquid.

#### 2.4.4. Effect of Elution Rate

Elution rate also is an important factor in dynamic desorption processes. As shown in Table S5 (Supporting Information), as the flow rate increased from 5BV to 15BV there was a gradual increase in the eluent of the contents of rutin and myricetin. When the flow rate continued to increase, it was accompanied by a decrease in the content of rutin and myricetin. For this reason, the elution rate at 15 BV/L was selected as the optimum desorption flow rate, which corresponded to the highest concentrations of rutin (164.32 μg/mL) and myricetin (189.12 μg/mL) in the desorption solution at that flow rate. In addition, rutin and myricetin, when eluted with 60% ethanol, reached a maximum concentration at about 5 BV (rutin, 65.32 μg/mL; myricetin, 43.96 μg/mL) and then gradually decreased to zero at 15 BV (Figure S5). Therefore, the optimal volume of eluent was selected as 15 BV.

### 2.5. Preparative Separation of FLAs from Goji Berries by Mixed-Mode MARs

**Figure 5:**
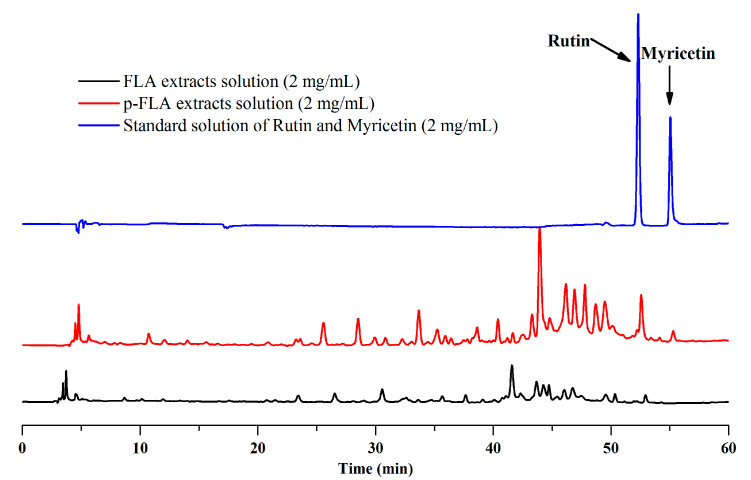
Chromatograms of flavonoid (FLA) extract, purified (p)-FLA extract solution, and standard solution of rutin and myricetin.

As described above, the optimal preparative separation conditions of FLAs by mixed-mode MARs were composed of AUKJ-1 and BWKX-1 in a 2:1 ratio, the loading concentration was 600 g/L, the flow rate was 10 BV/h, the elution rate was 15 BV/L, the eluent volume was 15 BV, and 60% ethanol was used as eluent. This method was applied to the separation and enrichment of FLAs from the supernatant named as the FLA extract solutions by the preparation of *Lycium barbarum* polysaccharide. After the dynamic adsorption process was absolutely completed at the optimum experimental condition with mixed-mode MARs, the obtained eluent solutions were analyzed by HPLC. According to the above experiments, the HPLC chromatograms of the FLA extract solutions and the purified FLA (p-FLA) solutions are shown in Figure 5. Compared to FLA extract solutions, the relative peak area and peak number of p-FLA solutions significantly increased after a one run treatment, and notably, the peak area of rutin increased by 6.7 times. It is worth mentioning that there was no peak of myricetin in FLA extract solutions, indicating that the content of myricetin in Goji berries was very low. However, after the enrichment with mixed-mode MAR E, the obvious peak of myricetin appeared in p-FLA solution. In addition, total flavonoids content of p-FLAs increased from 0.97% to 36.88% after one purification. As compared to other methods, this method possessed a lower yielding cost, was less labor intensiveness, had more procedural simplicity, and was more environmentally friendly. The results showed that this method could provide help in the development of large-scale production of high-purity FLAs from Goji berries in industry. Furthermore, the study may provide scientific references for separating and enriching other bioactive components from crude extracts of herbal raw materials using mixed-mode MAR E.

### 2.6. Anti-Aging and Antioxidant Effect of FLA Extract and p-FLA Extract in C. elegans

**Figure 6:**
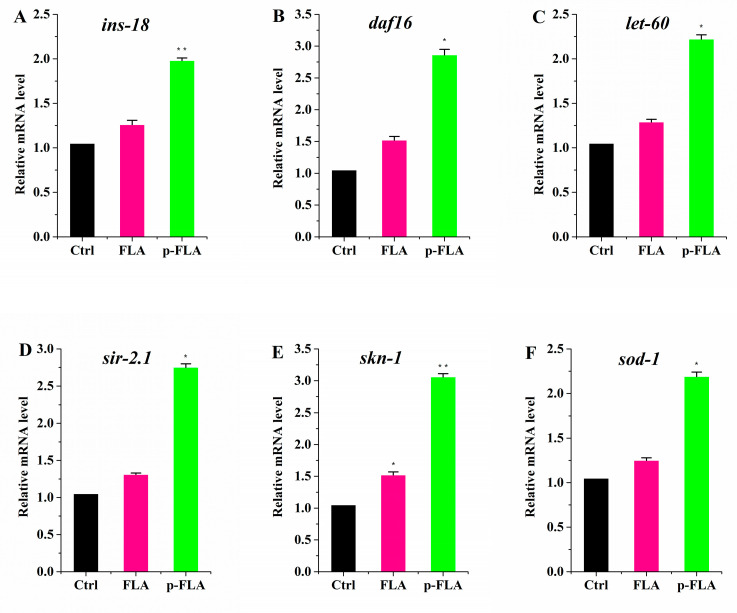
Anti-aging and antioxidant effect of FLA and p-FLA in *C. elegans*. (**A**)–(**D**): 2 mg/mL p-FLA extracts significantly increased the expression of *ins-18*, *daf-16*, *let-60* and *sir-2.1*, which are key anti-aging genes; (**E**): 2 mg/mL p-FLA extracts up-regulate *skn-1* expression; the effect was three times greater than FLA extracts; (**F**): 2 mg/mL p-FLA extracts increased the expression of antioxidant enzyme *sod-1* significantly. Error bars represent means ± SEMs (* *p* < 0.05, ** *p* < 0.01).

To evaluate the biological activity of FLA extract and p-FLA extract, the anti-aging and antioxidant effects of FLA and p-FLA were measured in the *C. elegans* model. After synchronization, the worms were cultured on normal NGM plates until young adults (Day 1), then transferred to the plates with FLA and p-FLA at a concentration of 2 mg/mL. When the worms were of age (on Day 12), total RNA was extracted and the expression of age-related genes was detected using qPCR. The data showed that p-FLA significantly increased the expression of *ins-18,* *daf-16,* *let-60* and *sir-2.1,* which are key anti-aging genes, while FLA had no influence on these genes’ expression compared to the control (Figure 6A–D). These results indicated that A had a better anti-aging effect in *C. elegans*. Further, the antioxidant activity of FLA and p-FLA were compared. *skn-1* is an important transcriptional factor initiating the antioxidant system. Although both FLA and p-FLA could up-regulate *skn-1* expression, the effect of p-FLA was more significant by about three times (Figure 6E,F). Furthermore, p-FLA could also increase the expression of antioxidant enzyme *sod-1,* suggesting that p-FLA had stronger antioxidant capacity.

### 2.7. Effect of FLA and p-FLA in Aβ-Expressing Nematode Model Strain CL2006

**Figure 7:**
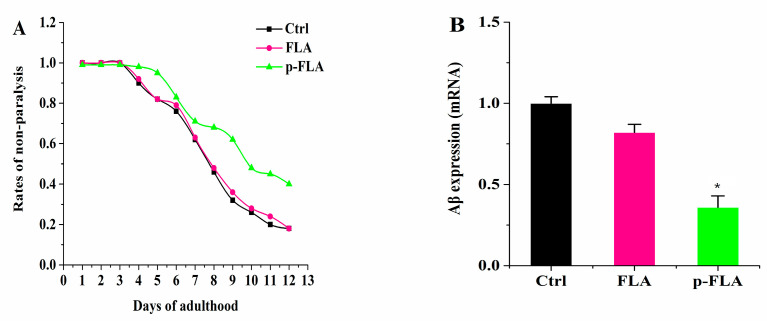
Effect of FLA and p-FLA in Aβ-expressing nematode model strain CL2006. (**A**): 2 mg/mL p-FLA extracts significantly delayed Aβ-induced paralysis in this transgenic worm from Day 1 to Day 12; (**B**): 2 mg/mL p-FLA extracts reduced the level of Aβ by half. Error bars represent means ± SEMs (* *p* < 0.05).

Alzheimer’s disease (AD) is widely recognized as a common aging-related neurodegenerative disorder. AD is thought to be caused by the production and deposition of neurotoxic Aβ-peptide in the brain. Therefore, the focus of research in toxic Aβ clearance is one approach for the treatment of AD. Therefore, in order to further evaluate the anti-aging effect of FLA and p-FLA, we used the Aβ-expressing nematode model strain CL2006 to investigate the effect of FLA and p-FLA. This transgenic nematode strain expresses the human 42 amino acid sequence of Aβ under the control of the muscle-specific unc-54 promoter of ***C. elegans*** and responds to Aβ expression with increased paralysis. The worms were treated with FLA and p-FLA at a concentration of 2 mg/mL from Day 1 to Day 12. Then, paralysis of the worms was analyzed, and total RNA was extracted to check the Aβ expression. From the results, p-FLA significantly delayed Aβ-induced paralysis in this transgenic worm, while FLA had no obvious influence (Figure 7A). Consistently, the level of Aβ was reduced by half with the treatment of p-FLA rather than FLA (Figure 7B). Therefore, in this AD model strain, p-FLA showed a better anti-aging effect.

## 3. Materials and Methods

### 3.1. Chemical Reagents and Solvents

Dried Goji berries were purchased from the local market of Ningxia Hui Autonomous Region in China. Analytical standard rutin (≥98%) and myricetin (≥98%) were purchased from Sigma-Aldrich (St. Louis, MO, USA). Ten different varieties of MARs were purchased from Cangzhou Bon Adsorber Technology Co., Ltd. (Cangzhou City, China), Cangzhou Yuanwei Chemical Industry Co., Ltd. (Cangzhou City, Hebei, China), and Xi’an Sunresin Technology Co., Ltd. (Xi’an, China). HPLC grade methanol was purchased from MREDA Technology Inc (Beijing, China). Double distilled water was prepared using a Milli-Q water purification system. All other chemicals were at least of analytical grade.

### 3.2. Apparatus

Centrifuges (TGL-15B) were from ShangHai Anting Scientific Instrument Factory (No. 400 Yuanda Road, Anting Town, Shanghai, China). The freeze dryer (10–B) was from Biosafer Company (Room 601, Building 2, No.108, East Ganjiabian, Yaohua Street, Qixia District, Nanjing, Jiangsu, China). Subcritical fluid extraction laboratory equipment (CBE-25L) was from Henan Subcritical Extraction Biological Technology Co., Ltd. (Shangsong Street 200m Honghe South High-tech Zone, Anyang, China). The high-speed shear dispersing emulsifier equipment (HSDE, T25) was from Werke GmbH & Co. KG (Janke & Kunkel-Str. 10 79219, Staufen, Germany). The HPLC equipment used was an Agilent 1200 HPLC system including a diode array detector (Agilent Technologies, 5301 Stevens Creek Blvd., Santa Clara, CA, USA).

### 3.3. Preselection of MARs

Ten different varieties of MARs, namely BSKB-1, BSKC-1, AUKJ-1, BWKS-1, BWKX-1, BMKX-4, BMKX-1, BMKX-3, BNKX-5, and BNKX-1 were selected according to the molecular dimensions and polarities of rutin and myricetin, as shown in Figure S2 (Supporting Information), to investigate the adsorption/desorption behaviors of them. Each variety of MARs was accurately weighed in weighing bottles on a Sartorius BT224S analytical balance, placed in a ZFD-5090 drying oven, and dried at 105 °C until a constant mass was obtained. The specific surface area and average pore size were calculated via the Brunauer–Emmett–Teller (BET) and Barrett–Joyner–Halenda (BJH) methods as previously reported [33]. The infrared spectra were obtained using Fourier-transform infrared spectrometry (FTIR) with a spectrophotometer (Thermo Nicolet, NEXUS, Waltham, MA, USA) via the potassium bromide technique in the range of 400 cm^−1^ to 4000 cm^−1^. The measured parameters are depicted in Table S1 (Supporting Information).

### 3.4. Preparation of FLAs Solutions and Sample Solutions

Dried Goji berries were smashed into plant powder using a grinder (FW100, Taisite Instrument Co., Ltd., Tianjin, China); then the powder (1 kg) was extracted via subcritical fluid extraction laboratory equipment with butane to remove oil and colored components. The pretreated powder was immersed in 12 L of distilled water and then heated to 60 °C; after immersing the rotor of the HSDE machine under the aqueous solution, the switch was turned on, and the rotation was set at 15,000 rpm and the extraction time to 60 min. After extraction, the obtained extracts were centrifuged at 8000 rpm for 15 min, and the supernatant was collected and pooled as final extracts for further concentrated under reduced pressure to obtain a final volume of 2 L. Then 4 volumes of 95% EtOH were added under vigorous stirring, and the mixture was left to stand overnight at 4 °C, after which it was centrifuged, and the supernatant was dried to obtain the dry FLA extract. Finally, the extract was diluted with deionized water to obtain 600 mg/mL actual FLA solution. Then, 25 mg of rutin was accurately weighed and dissolved in 25 mL ethanol solution to obtain a rutin stock solution with a concentration of 1 mg/mL; additionally, 1 mg/mL myricetin stock solution was obtained by the same method as above. All sample solutions for HPLC determination were filtered through a 0.45 μm nylon membrane before entering the liquid phase.

### 3.5. HPLC-DAD Analysis of Rutin and Myricetin

Rutin, myricetin, actual FLAs, and purified FLA extracts were quantified by an Agilent 1200 series HPLC system (Agilent Technology, USA). The analysis was performed on a reversed-phase C18 column (250 mm × 4.6 mm, I.D., 5 μm), maintained at 25 °C. The mobile phase contained acetonitrile (A) and 5% acetic acid solution (B), and the gradient elution of the mobile phase was as follows: 3–10% (B) in 0–0 min, 10–15% (B) in 10–20 min, 15–25% (B) in 20–30 min, 25–40% (B) in 40–50 min, and 40–70% (B) in 50–60 min. The flow rate was maintained at 1.0 mL/min throughout the analysis. The injection volume was 20 μL for each sample. The wavelength of the diode array detector was selected as 280 nm.

### 3.6. Static Adsorption/Desorption Experiments

#### 3.6.1. Static Adsorption/Desorption Experiments of Ten Resins

The static adsorption/desorption experiments with rutin and myricetin were carried out as follows: pre-weighed amounts of resins among the 10 resins (equivalent to 1.2 g dry resin) and 20 mL rutin or myricetin solution were mixed together in a 250 mL stoppered-conical flask. The flasks were shaken continually in a SHA-B shaker–incubator (100 rpm) for 4.5 h at 25 °C. The final concentrations of rutin or myricetin solutions were analyzed by HPLC. The static desorption experiments were carried out as follows: after the adsorption equilibrium was reached, the adsorbate laden resins were desorbed with 100 mL 60% ethanol solution, and the flasks were shaken (100 rpm) for 12 h at 25 °C. The corresponding concentrations of rutin and myricetin in solutions were quantified with HPLC.

#### 3.6.2. Static Adsorption/Desorption Experiments of Mixed-Mode Resin

According to the adsorption and desorption characteristics of ten kinds of macroporous adsorption resins for rutin and myricetin, two optimal resins were identified. Then the mixed-mode resin were built with these two resins in different proportions (1:1, 1:1.5, 1:2, 1.5:1, 2:1). The static adsorption and desorption effect of the mixed-mode resin was evaluated according to the method of Section 3.6.1. Both adsorption/desorption ratio and adsorption/desorption capacities of each individual resin and mixed-mode resins was calculated according to the following Equations (1)**–**(4):

Adsorption ratio:

(1) $$A\left(\%\right)=\frac{\left(C_{0}-C_{e}\right)}{C_{0}}\times 100\%$$

Adsorption capacity:

(2) $$q_{e}=\left(C_{0}-C_{e}\right)\times \frac{V_{i}}{\left(1-M\right)W}$$

where *A* is the adsorption ratio (%), and q_e_ is the adsorption capacity (mg/g dry resin) at the adsorption equilibrium. *C_0_* and *C_e_* are the initial and equilibrium concentrations of rutin and myricetin in sample solutions, respectively (mg/L). *M* is the moisture content of the resin (%). *W* is the weight of the resin used (g). *V_i_* is the volume of sample solutions used in this study (mL).

Desorption ratio:

(3) $$D\left(\%\right)=C_{d}\times \frac{V_{d}}{\left(C_{0}-C_{e}\right)V_{i}}\times 100\%$$

Desorption capacity:

(4) $$q_{d}=C_{d}\times \frac{V_{d}}{\left(1-M\right)W}$$

where *D* is the desorption ratio (%), and *q_d_* is the desorption capacity (mg/g dry resin) after desorption equilibrium. *C_d_* is the concentrations of rutin and myricetin in the desorption solution (mg/L). *V_d_* is the volume of the desorption solution (mL). *C_0_, C_e_, M, W,* and *V_i_* are the same as defined above.

### 3.7. Dynamic Adsorption/Desorption Experiments

Dynamic adsorption/desorption experiments were performed in a lab-scale glass column (inner diameter 1.2 cm, length × 27 cm) packed with 10 g AUKJ-1 resin and 5 g BWKX-1. The bed volume (BV) of the wet-packed resin was 5 mL, and the length of the packed resin bed was 12 cm. The adsorption process was initiated by loading the sample solution into the pretreated glass column. The adsorbate-laden column was washed with distilled water and then desorbed with 60% ethanol solution. An aliquot of the eluent was directly analyzed by HPLC. The eluent was further concentrated using a rotary evaporator, dried under vacuum, and the purity of the product was calculated. Several variables, such as feeding concentration and flow rate during the adsorption process, the composition of ethanol-aqueous solutions (***v/v***), eluent volume, and flow rate during the desorption process were systematically investigated. Then, the actual FLAs extract was purified to obtain the purified FLA extracts under optimal conditions.

### 3.8. Caenorhabditis elegans Strains and Treatment

The *C. elegans* strains used in this study were Bristol N2 and CL2006. The Bristol N2 strain was obtained from the Caenorhabditis Genetics Center (CGC) at the University of Minnesota, USA. The CL2006 strains were gifts from Hong Zhang’s lab at the Chinese Academy of Sciences. All strains were maintained at 20 °C on nematode growth medium (NGM) seeded with the *Escherichia coli* OP50 feeding strain. FLA and p-FLA were added into the NGM growth medium at the concentration of 2 mg/mL. The worms were given the treatment from young adults (Day1), and the treatment lasted for 12 days.

### 3.9. Worm Synchronization and Paralysis Assay

Worm synchronization was implemented by alkaline hypochlorite treatment of gravid adults. Worms were washed with M9 buffer (3 g of KH_2_PO_4_, 6 g of Na_2_HPO_4_, 5 g of NaCl, 1 mL of 1 mol/L MgSO_4_, in H_2_O to 1 L) and pelleted by centrifugation (2000 g). Then, the worms were incubated in hypochlorite solution (1 mL of 2 N NaOH, 800 μL of sodium hypochlorite solution, 2.2 mL of dH_2_O) for 3–5 min to homogenize the large worm particles. Eggs were pelleted by centrifugation and washed at least three times with M9 buffer; then, they were incubated in M9 buffer and allowed to hatch overnight at 20 °C. The synchronized L1-stage worms were put on standard NGM plates coated with OP50 at 20 °C. To identify paralysis, each worm was gently touched with a platinum loop. The worm was considered paralyzed if it did not move or moved only its head after being touched. The worms were tested for paralysis every day.

### 3.10. Real-Time qPCR

About one thousand worms in each group were used to extract total RNA using TRIzol reagent according to the manufacturer’s (Invitrogen) protocol. RNA samples were then reverse transcribed using M-MuLV reverse transcriptase (Promega), and mRNA levels were measured by performing RT-qPCR on a 7500 Real-Time PCR System (Applied Biosystems, Waltham, MA, USA). The samples were heated to 95 °C for 2 min and subjected to 40 cycles of amplification (1 min at 94 °C, 1 min at 58 °C, and 1 min at 72 °C), followed by 10 min at 72 °C for the final extension.

## 4. Conclusions

As is well known, the method of employing mixed-mode MARs to separate active ingredients from different herbal extracts in traditional Chinese medicine has attracted increasing attention due to many special advantages such as simple operation process, low operation cost, little solvent consumption, high efficiency, easy regeneration, long lifetime, and environmental friendliness. In this paper, the mixed-mode MARs for purification of FLAs from *Lycium barbarum* L. through computer-assisted calculation of the molecular size of flavonoids and the precise matching of MAR physical and chemical properties was firstly developed. By simulating the molecular size of rutin and myricetin, which are major flavonoids present in *Lycium barbarum* L., ten different varieties of MARs of suitable molecular dimensions were used to investigate the adsorption/desorption behaviors of flavonoids from *Lycium barbarum* L. extracts. Both AUKJ-1 and BWKX-1 showed higher separation efficiency than other MARs and then were mixed in a 2:1 ratio to constitute a mixed-mode MAR E to obtain the optimal adsorption efficiency. Under optimal separation conditions including feeding concentration at 600 g/L, flow rate at 10 BV/h, elution rate at 15 BV/L, and 60% ethanol as eluent, the content of flavonoids from *Lycium barbarum* L. increased by 38-fold from 0.97% to 36.88% after one purification. The refined flavonoids had a better anti-aging effect and stronger antioxidant capacity in *C. elegans*, which could significantly increase the expression of the anti-aging genes *ins-18*, *daf-16*, *let-60* and *sir-2.1*, as well as up-regulate *skn-1* and *sod-1* expression. In addition, the refined flavonoids played an important role in aging-related Alzheimer’s disease by down-regulating Aβ expression. This article may offer a suitable method for large-scale separation and purification of flavonoids and provide novel ideas for food processing or pharmaceutical industries.

## Supplementary Materials

The following are available online at https://www.mdpi.com/1420-3049/25/15/3511/s1, Figure S1: Chemical structure of flavonoids from Goji berries; Figure S2: The molecular dimensions and logP of rutin and myricetin; Figure S3: Adsorption capacities for rutin and myricetin on ten resins; Figure S4: Desorption capacity of rutin and myricetin on five different ratio resins (A–E: AUKJ-1 and BWKX-1 in1:1, 1:1.5, 1.5:1, 1:2, 2:1 in turn); Figure S5: Effect of eluent volume on the desorption capacity of rutin and myricetin. Table S1: Physical properties and the moisture contents of the MARs used; Table S2: Effect of loading concentration on the adsorption capacity of rutin and myricetin; Table S3: Effect of loading rate on the adsorption capacity of rutin and myricetin; Table S4: Effect of ethanol concentration on the desorption capacity of rutin and myricetin; Table S5: Effect of elution rate on the desorption capacity of rutin and myricetin.
