# Supplementary material for: Preparative Separation of Flavonoids from Goji Berries by Mixed-Mode Macroporous Adsorption Resins and Effect on Aβ-Expressing and Anti-Aging Genes
Source: Molecules. 2020 Jul 31;25(15):3511. doi: 10.3390/molecules25153511 (PMC7435390; doi:10.3390/molecules25153511)
Supplement: Supplementary file 1 [file molecules-25-03511-s001.pdf]

molecules

SUPPLEMENTARY MATERIALS

Preparative Separation of Flavonoids from Goji Berries by Mixed-Mode Macroporous Adsorption Resins and Effect on Aβ-Expressing and Anti-Aging Genes

Jianfei Liu [1,2,†], Jiao Meng [3,†], Jinhao Du [4], Xiaofeng Liu [4], Qiaosheng Pu [5], Duolong Di [1,2,*] and Chang Chen [2,3,*]

1 CAS Key Laboratory of Chemistry of Northwestern Plant Resources and Key Laboratory for Natural Medicine of Gansu Province, Lanzhou Institute of Chemical Physics, Chinese Academy of Sciences, Lanzhou 730000, China; jfliu@licp.cas.cn (J.L.)
2 University of Chinese Academy of Sciences, Beijing 100049, China
3 National Laboratory of Biomacromolecules, CAS Center for Excellence in Biomacromolecules, Institute of Biophysics, Chinese Academy of Sciences, Beijing 100101, China; mengjiao@ibp.ac.cn (J.M.)
4 National Laboratory of Biomacromolecules, Institute of Biophysics, Chinese Academy of Sciences, Beijing 100101, China; djh_8122069@163.com (J.D.); 18009400938@163.com (X.L.)
5 State Key Laboratory of Applied Organic Chemistry, Key Laboratory of Nonferrous Metals Chemistry and Resources Utilization of Gansu Province, Department of Chemistry, Lanzhou University, Lanzhou 730000, China; puqs@lzu.edu.cn (Q.P.)
* Correspondence: didl@licp.cas.cn (D.D.); changchen@moon.ibp.ac.cn (C.C.); Fax: +86-931-4968248 (D.D.); +86-010-64888406 (C.C.)
† These authors contributed equally to this work.

Received: 30 May 2020; Accepted: 21 July 2020; Published: date

SUPPLEMENTARY MATERIALS CONTENT

Figure S1. Chemical structure of Flavonoids from Goji berries;
Figure S2. The molecular dimensions and logP of Rutin and Myrucetin;
Figure S3. Adsorption capacities for Rutin and Myricetin on ten resins;
Figure S4. Desorption capacity of Rutin and Myricetin on five different ratio resins (A~E: AUKJ-1 and BWKX-1 in1:1, 1:1.5, 1.5:1, 1:2, 2:1 in turn);
Figure S5. Effect of eluent volume on the desorption capacity of Rutin and Myricetin.
Table S1. Physical properties and the moisture contents of the MARs used;
Table S2. Effect of loading concentration on the adsorption capacity of Rutin and Myricetin;
Table S3. Effect of loading rate on the adsorption capacity of Rutin and Myricetin;
Table S4. Effect of ethanol concentration on the desorption capacity of Rutin and Myricetin;
Table S5. Effect of elution rate on the desorption capacity of Rutin and Myricetin.

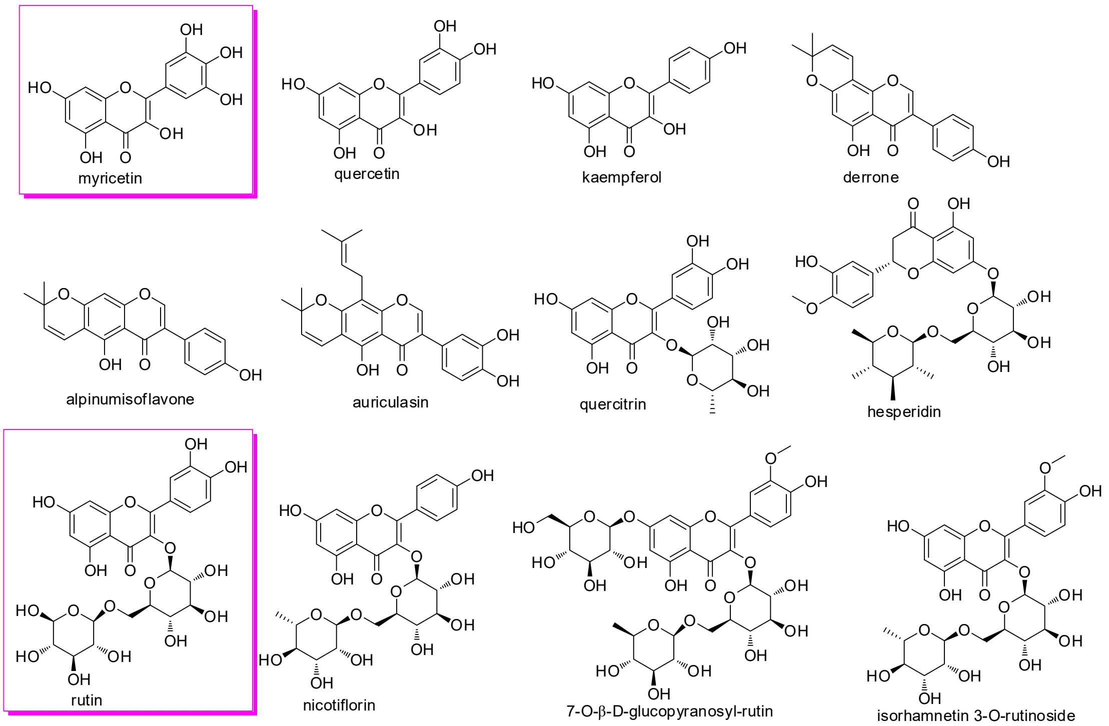

Figure S1. Chemical structure of Flavonoids from Goji berries.

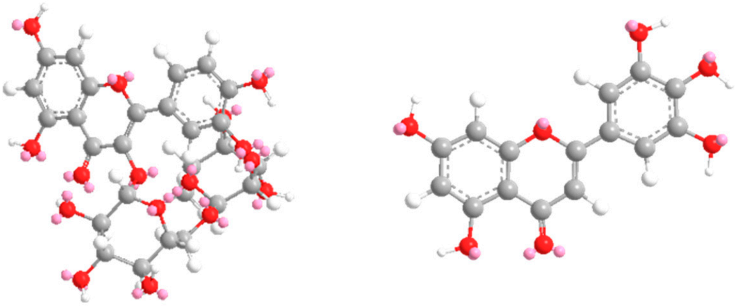

Molecular dimensions is 1.382 nm and logP is -5.91

Rutin

Molecular dimensions is 1.135 nm and logP is -5.04

Myricetin

Figure 2. The molecular dimensions and logP of Rutin and Myrucetin.

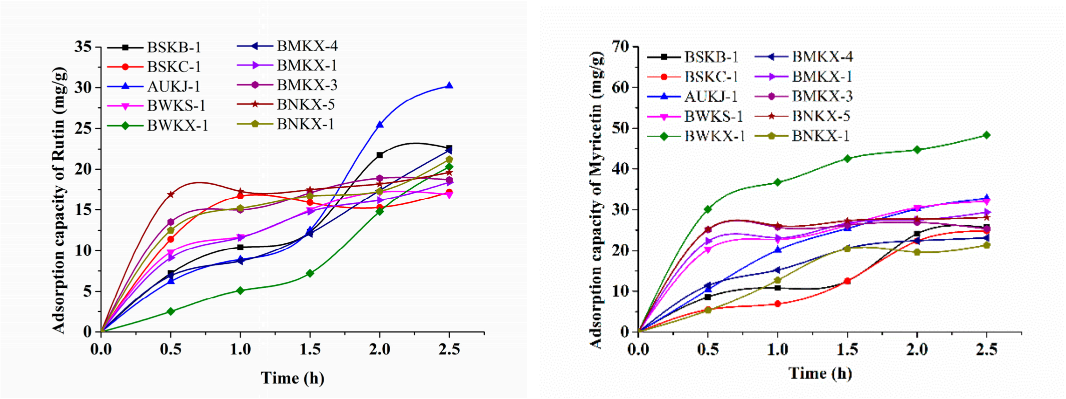

Figure 3. Adsorption capacities for Rutin and Myricetin on ten resins.

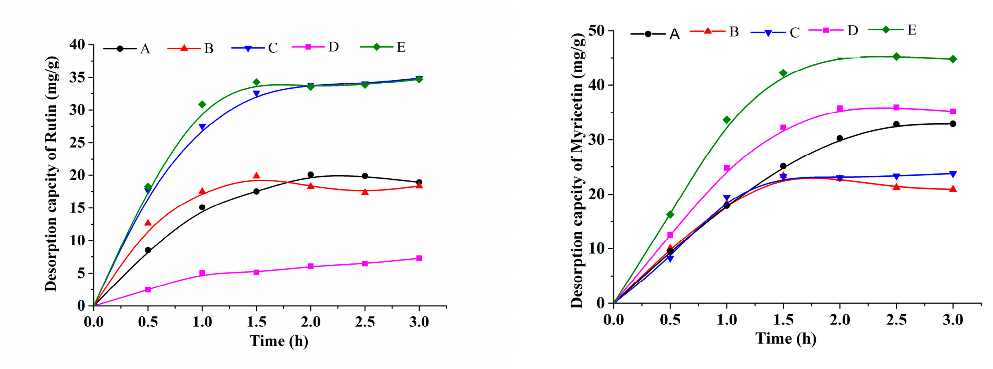

Figure 4. Desorption capacity of Rutin and Myricetin on five different ratio resins (A~E: AUKJ-1 and BWKX-1 in1:1, 1:1.5, 1.5:1, 1:2, 2:1 in turn).

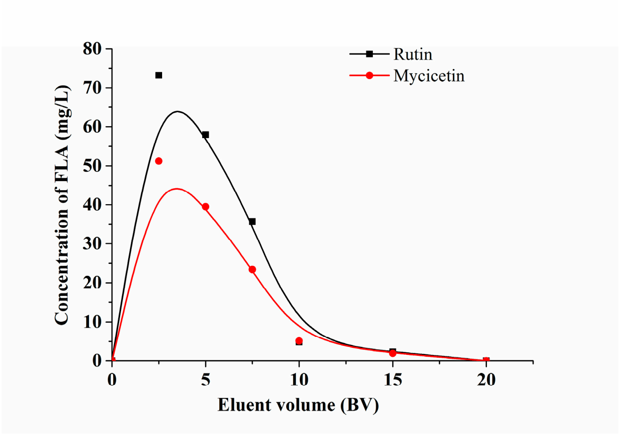

Figure 5. Effect of eluent volume on the desorption capacity of Rutin and Myricetin.

Table 1. Physical properties and the moisture contents of the MARs used.

Resins series	Polarity	Structure	Specific Surface Area (m^2/g)	Pore Size (nm)	Moisture Content
BSKB-1	Strong	SDVB	550-600	8.0	74.34%
BSKC-1	Strong	SDVB	650.4	8.0	53.82%
AUKJ-1	Weak	SDVB	1326.9	2.32	45.08%
BWKS-1	Weak	SDVB	600.0	11	57.53%
BWKX-1	Weak	SDVB	985.7	5.37	72.95%
BMKX-4	Middle	SDVB	658.0	7.25	65.61%
BMKX-1	Middle	SDVB	562.1	7.18	46.59%
BMKX-3	Middle	SDVB	656.9	3.49	56.72%
BNKX-5	Non	SDVB	735.7	7.52	70.53%
BNKX-1	Non	SDVB	900.0	6.25	59.31%

Table 2. Effect of loading concentration on the adsorption capacity of Rutin and Myricetin.

Concentration (g/L)	400	600	800	1000	1200
Rutin (μg/mL)	40.10	41.06	60.98	94.89	140.90
Myricetin (μg/mL)	6.91	7.57	34.57	37.58	39.10

Table 3. Effect of loading rate on the adsorption capacity of Rutin and Myricetin.

Flow rate (BV/h)	5	10	15	20	25
Rutin (μg/mL)	109.41	95.68	152.44	195.43	291.51
Myricetin (μg/mL)	48.84	16.70	86.42	108.06	127.48

Table 4. Effect of ethanol concentration on the desorption capacity of Rutin and Myricetin.

**Ratio of ethanol (%)**	**30**	**45**	**60**	**75**	**90**
Rutin (μg/mL)	87.81	244.55	503.03	456.51	382.16
Myricetin (μg/mL)	102.3	229.85	549.25	434.95	180.35

Table 5. Effect of elution rate on the desorption capacity of Rutin and Myricetin.

**Flow rate (BV/h)**	**5**	**10**	**15**	**20**	**25**
Rutin(μg/mL)	134.54	148.43	164.32	135.38	109.90
Myricetin(μg/mL)	101.66	146.26	189.12	183.30	177.45
